# Sensory and Volatile Compounds Characteristics of the Sauce in Bean Paste Fish Treated with Ultra-High-Pressure and Representative Thermal Sterilization

**DOI:** 10.3390/foods12010109

**Published:** 2022-12-25

**Authors:** Jie Zhao, Yimao Zhang, Yu Chen, Yuhui Zheng, Changbo Peng, Hongbin Lin, Zhenming Che, Wenwu Ding

**Affiliations:** 1 College of Food and Bioengineering, Xihua University, Chengdu 610039, China; 2 Chongqing Key Laboratory of Speciality Food Co-Built by Sichuan and Chongqing, Chongqing 400000, China

**Keywords:** condiment, volatile composition, sterilization, sensory evaluation, GC×GC-MS

## Abstract

This study investigated the differences between three sterilized samples to reveal the unique aroma characteristics of the sauce in bean paste fish by multiple analysis methodologies. Samples were subjected to pasteurized (PS), high-temperature sterilization (HTS), and ultra-high-pressure treatment (UHP) tests. The UHP had a higher sensory evaluation and could better maintain the original flavor of the sample. A total of 92, 83, 85, and 76 volatile compounds were detected via comprehensive two-dimensional gas chromatography-mass spectrometry (GC×GC-MS) techniques in the control (CT), PS, HTS, and UHP groups, respectively. According to the analysis of gas chromatograph-olfactometry and odor activity value, 7 compounds were considered to have an aromatic influence on the sauces, in which four compounds (1,8-Cineole, Linalool, Hexanal, and Dimethyl trisulfide) exhibited a positive contribution to the aroma of the sauces. PLS-DA results showed that the UHP group positively correlated with volatiles (Isoamylol and 1-Octen-3-ol), color, and gloss. In general, the UHP treatment could retain the original state and flavor of the sauce, showing a high similarity to the control group. The HTS significantly altered the flavor and status of the samples.

## 1. Introduction

Pixian Douban (PXDB), a traditional Chinese fermented condiment, is one of the important ingredients in compound seasonings, such as the sauce of bean paste fish (a famous compound condiment of Sichuan cuisine). This sauce was made from materials including PXDB, pickled chili pepper, ginger, garlic, and rapeseed oil after frying and obtained an instant seasoning for bean paste fish. With the industrialization of the traditional favorites, the sauce has been put into production, in which the flavor and taste might alter during sterilization or other processing treatment, causing the taste or aroma of the sauce to fluctuate [1]. It is known that the flavor of sauces plays a crucial role in the quality and acceptability of food. Many research studies have estimated the characterization of volatile components in sauces after processing [2,3,4].

Sterilization is widely used to ensure product safety and extend shelf life, although it may alter the original aroma profile of sauces. Thermal sterilization, with its advantages of solid practicability, easy operation, high efficiency, and low cost, is an essential standard procedure in the food industry [5,6]. However, this treatment may lead to quality changes (mainly in appearance and nutrition) of sauce, forming odors that seem ‘off’ and losing desirable aromas. The freshly-cooked sauces were preferred by consumers due to the greater acceptability of flavor, color, and taste. Thus, exploring an alternative to thermal sterilization for preservation of the sauce with original quality and flavors has gained more attention among the food industry and researchers. Moreover, non-thermal sterilization technology has been increasingly used in scientific research in recent years because it can reduce or eliminate the adverse effects of high temperatures [7].

As an alternative to thermal sterilization, ultra-high-pressure (UHP) sterilization is considered one of the most promising methods [8,9]. UHP technology has been widely applied in meat dishes [10,11,12]. Recent studies found that high-pressure processing could meet consumer demand in maintaining food quality and heat-sensitive nutrients, as well as in formulating clean-label products with minimal or no synthetic chemicals [13]. Sun et al. [4] discovered that the flavor components of sweet and sour pork were affected by different sterilization treatments, and that ultra-high-pressure treatment might improve the taste quality of the dishes. Compared to the ultra-high-pressure treatment, the quality of green chili paste had changed obviously in the heat treatment group, owing to the Maillard reaction [14]. Moreover, UHP was often applied in juice sterilization, which presented distinct advantages in maintaining the primordial quality or aroma characteristics of juice [8,9,15,16]. For sauces, the aroma compounds readily altered during the sterilization treatment, while limited studies examined the changes of volatile components in sauces. Therefore, this study focused on the variation of volatile compounds in sauces during sterilization, providing a convenient method to understand the actual sensory changes of sauce after different sterilization processes and offering a theoretical basis for sterilization of the sauce in bean paste fish.

Therefore, this study mainly characterized the sensory and volatile compounds of the sauce in bean paste fish treated with pasteurization (PS), high-temperature sterilization (HTS) and ultra-high-pressure treatment (UHP). The sensory properties and volatile compounds are discussed to evaluate the impact of sterilization on the flavor characteristics of the sauce. In addition, the relationships between sensory attributions and aroma-active compounds were identified. A deep understanding of aroma changes during sterilization may contribute to the large-scale production of sauces in bean paste fish.

## 2. Materials and Methods

### 2.1. Chemicals

The glutinous rice black vinegar was obtained from Qianhe Condiment And Food Co., Ltd. (Sichuan, China). The pickled chili pepper was obtained from Sichuan Guangle Food Co., Ltd. (Chengdu, China). The Great Value sweet potato starch was obtained from Walmart Hypermarket (Chengdu, China). The chicken essence was obtained from Sichuan Guosha Industrial Co., Ltd. (Chengdu, China). The Fortune pressed canola oil was obtained from COFCO Corporation (Beijing, China). The white granulated sugar was obtained from Yunnan Dianwang Yi Agricultural Technology Development Co., Ltd. (Yunnan, China). Garlic and ginger were purchased at a local agricultural product market.

### 2.2. Sample Preparation

**Figure 1:**
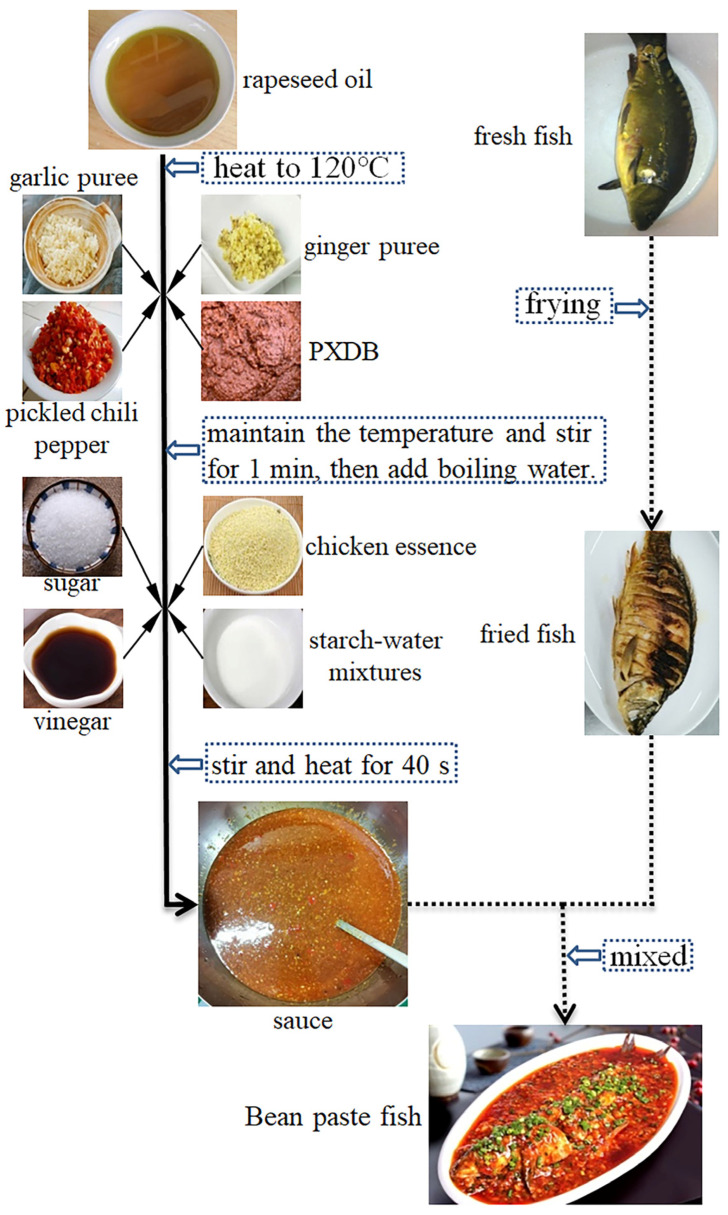
The simplified process of making bean paste fish.

The PXDB was prepared based on our previous study using a closed fermentation system under gradient steady-state temperature field [17]. As shown in Figure 1, the sauce was prepared using PXDB and other ingredients. Briefly, PXDB (15%), ginger puree (8.5%), garlic puree (8.5%), and pickled chili pepper (24%) were added into rapeseed oil (26%) when it was heated at 120℃, maintaining the temperature and stirring for 1 min. Then, boiling water as well as sugar (0.4%), chicken essence (0.4%), starch-water mixtures (water 8.5%, starch 6.7%), and vinegar (2%) were added, stirred and heated for 40 s. After that, the samples were sterilized by pasteurized (PS, 90 °C, 101 KPa, 10 min), high-temperature sterilization (HTS, 121 °C, 0.12 MPa, 10 min), and ultra-high-pressure (UHP, 25 °C, 500 MPa, 10 min) treatments, respectively. The fresh sauce was used as the control group (CT). Samples were stored at −18 °C for one week for further analysis.

### 2.3. Quantitative Descriptive Analysis (QDA)

The sensory methodology was applied to evaluate the overall taste and texture properties of sauces, in which the aroma flavor was not involved and it was detected by the objective instruments. Assessors were chosen based on the previous report with minor modification [16]. Students from the College of Food and Bioengineering in Xihua University were recruited as candidates. An initial recruitment questionnaire was used to screen candidates for their sensory understanding and familiarity with sauces and to determine their health status. Next, they were required to complete sensory ability tests for aroma identification and ranking in the allotted time. After that, they were ranked according to their test scores, and the top 25 candidates were selected. Assessors were chosen from candidates who scored at least 70% acuity on the sensory ability tests.

Quantitative descriptive analysis (QDA) was performed by 12 selected assessors (6 males and 6 females, 24 years old on average), who were experienced and familiar with the aroma of sauce. The evaluation method was based on the procedure of Xiao et al. [18] with minor modifications. First, each sensory assessor proposed their own sensory descriptors for the sauces, and after discussion the sensory descriptors were ascertained if more than half the assessors agreed. In our study, the descriptors were determined including pungency, acidity, color, gloss, saltiness, viscosity, and fluidity. Then, the sensory assessors were trained to perform sensory evaluation of the sauce test standards until all sensory assessors were able to correctly identify the test standards. Two grams of the sauce was placed in a 30 mL plastic cup coded with a random three-digit number and assessed using a ten-point scale (0 = none, 10 = very strong) at room temperature (23 ± 2 °C). The sensory evaluations of each sample were carried out in triplicate, and the results were averaged for each aroma attribute. All sensory evaluation tests were conducted in a standard sensory room, with each assessor tested in a separate compartment and mouth rinses with purified water (37 °C) before and after each evaluation.

### 2.4. Characterizing the Volatile Compounds

The electronic nose was first applied to roughly analyse the discrimination of volatile flavor substance between samples. Then, the flavor substances were extracted and identified in detail via HS-SPME\GC×GC-MS method. In addition, the differences of the key flavor substances between samples were further determined.

#### 2.4.1. E-Nose Analysis

A portable E-nose (PEN3, Win Muster Airsense Analytics Inc., Schwerin, Germany) was used to analyze the volatile compounds. Before testing, the e-nose was warmed up and calibrated with processed pure air to ensure data stability. An aliquot of 2 g sample was placed into a 30 mL closed glass vial, equilibrated at 35 °C for 30 min. The parameters of the electronic nose were as follows: flow rate of 500 mL/min, measurement time of 90 s, interval time of 1 s, rinsing time of 120 s, and automatic zeroing time of 5 s. Each sample was measured at least three times.

#### 2.4.2. Extraction of Volatile Compounds

The volatile compounds of samples were determined by the solid-phase microextraction method (SPME) [19]. The mixture of n-alkanes (C7-C25, ≥99%) and 1,2-dichlorobenzene (as the internal standard, 99%) was obtained from Sigma-Aldrich Co., Ltd. (Shanghai, China). Briefly, about 5.0 g sample was taken into a 15 mL headspace vial where 10 μL 1,2-Dichlorobenzene (100 μg/mL in methanol) was added as the internal standard. Then, the vial was sealed and equilibrated at 55 °C for 45 min. Afterward, a carboxen-polydimethylsiloxane fused silica (CAR/PDMS, 75 μm) coating fiber (Supelco, Inc., Bellefonte, PA) was inserted into the headspace of the vial to absorb the volatiles at 55 °C for 45 min. GC injectors were used for desorption.

#### 2.4.3. Identification of Volatile Compounds by Comprehensive Two-Dimensional Gas Chromatography

Volatile compounds were analyzed by GC×GC-MS (QP2020; Shimadzu Co., Kyoto, Japan) equipped with a DB-Wax (polar; 36.4 m × 0.25 mm × 0.25 μm; Agilent Technologies Inc., Santa Clara, CA, USA) and DB-17 ms columns (non-polar; 1.2 m × 0.18 mm × 0.18 μm; Agilent Technologies). The GC×GC-MS operation was carried out according to the report, with some modifications made to the method [3]. A 40 °C initial column temperature was maintained for 2 min, followed by a 5 °C/min increase to 230℃ for 4 min. The same GC oven was used for both columns. The mass spectrometer was operated in electron impact mode with the electron energy set at 70 eV and a scan range of 41-330 m/z. Both columns were heated and cooled using a solid-state modulator SSM1800 (J&X Technologies Co., Ltd., Shanghai, China). The temperatures for hot zone entry and exit were 70 °C and 160 °C offsets relative to the oven temperatures, respectively, with a cap temperature of 260 °C and 320 °C for entry and exit hot zones [20]. This modulation period was set at 4 s and the cold zone temperature at 51 °C. Retention indices [14] were calculated with the C7–C30 n-alkane series (≥99%; Sigma-Aldrich Co., Ltd., Shanghai, China) under the same chromatographic conditions. Comparing the mass spectra of the volatile compounds with those in the National Institute of Standards and Technology library (NIST17) and previous literature allowed identification. Each group consisted of at least three parallel samples.

#### 2.4.4. Determination of Aroma-Active Components by GC-O

A gas chromatograph equipped with an olfactory detector (GC-O, Shimadzu) was employed to analyze the aroma-active compounds. The sample was injected into the splitless mode at 200 °C for 5 min. The temperature of the oven was adjusted to match that of GC×GC-MS. After the sample injection, three trained assessors recorded retention times and descriptions of aromas (replaced at 10 min intervals). The samples were sniffed by each trained assessor and each sample was measured at least three times.

For volatile compounds, their OAV value was calculated based on the concentration of the compound and their corresponding odor threshold in water obtained from the literature [21]. The sauce of bean paste fish was usually thought to be scented by compounds with OAVs >1. Equation (1) was as follows:

(1) $${\mathrm{OAV}}_{i}=\frac{\mathrm{the}\;\mathrm{concentration}\;\mathrm{of}\;\mathrm{the}\;\mathrm{compound}\;i}{\mathrm{odor}\;\mathrm{threshold}}$$

### 2.5. Statistical Analysis

All analyses were conducted with three parallel tests. Data was reported as a mean concentration ± standard deviation. The diagrams were conducted by Origin 2021 (OriginLab Inc., Northampton, MA, USA). Principal components analysis (PCA) of electronic nose analyses and partial least squares discriminant analysis (PLS-DA) of volatiles indexes were applied by SIMCA 14.1 (Umetrics, Umea, Sweden).

## 3. Results and Discussion

### 3.1. Quantitative Descriptive Analysis (QDA) of Sauces

**Figure 2:**
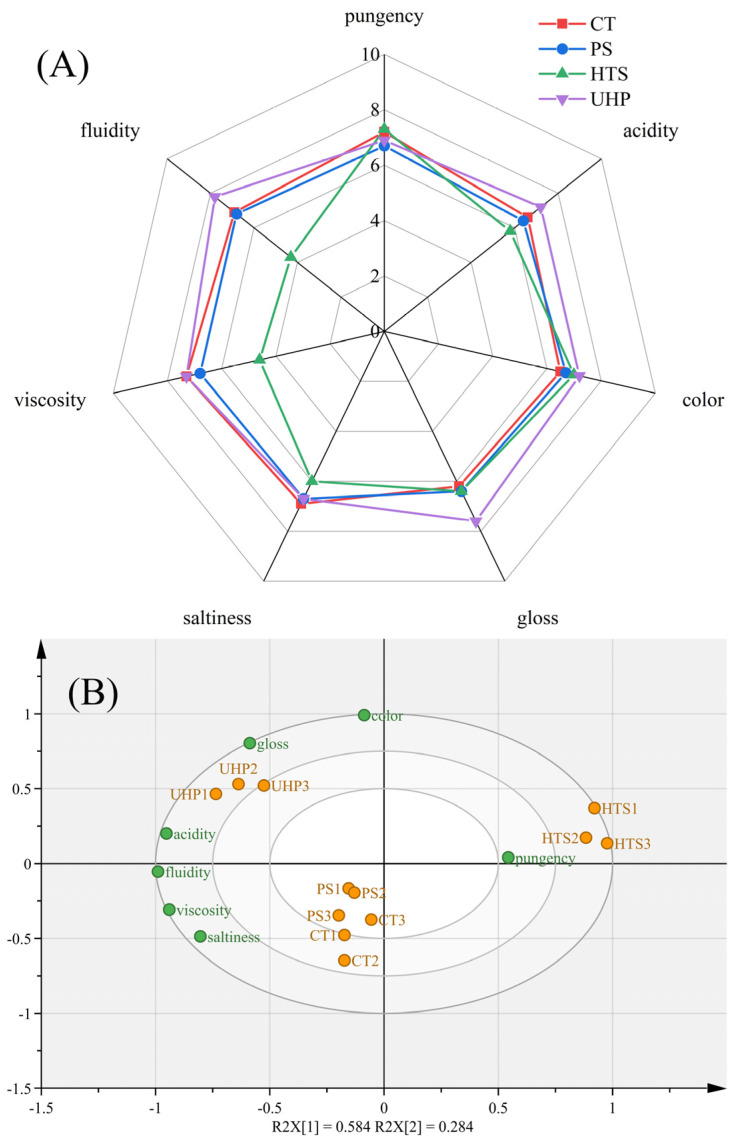
Sensory characteristics of the sauce by different sterilization methods (**A**) Spider web diagram; (**B**) PCA bi−plot generated from the sensory descriptors (*p* < 0.05).

QDA was performed to distinguish the sensory quality under different sterilization methods (Figure 2A). In terms of sensory evaluation scores, the CT group was used as a criterion to contrast how the sterilization treatments affected the flavor of the samples. Among the experimental groups, the PS group showed a similar profile to the CT group, and the UHP group showed the highest sensory composite score, indicating that the UHP treatment resulted in the best sauce quality. Mastello et al. [22] also elucidated that orange juice presented a similar flavor profile to fresh juice after UHP treatment. In contrast, the HTS group differed significantly from the CT group, suggesting that part of the sensory qualities could be negatively impacted by the high temperature.

As shown in Figure 2A, the PS group observed a slight decrease in pungency and viscosity compared to the CT group. At the same time, the other five evaluation scores in the PS sample were almost identical to the CT group. The viscosity scores in the HTS group decreased to 4.6 because of the disintegrating of gelatinized starch in the heating process. Significant changes were observed in fluidity after HTS sterilization, which were 6.9 for control and 4.3 for HTS, respectively. Secondly, the salinity and acidic quality in the HTS group were distinctly reduced comparing with the CT group, and this may attribute to the degradation of flavorful substances in the sauce. In contrast, the score of color in the HTS group was slightly higher than the control group. This could relate to the Maillard reaction, which occurs at high temperatures and generates a brown product to deepen the color. For the UHP group, the scores of the viscosity, saltiness, and pungency indicators were practically the same as those of the CT group. A significant increase was observed in the scores of the other four indicators (fluidity, acidity, color, and gloss) in the UHP group, indicating that UHP could improve the original quality of the sauce.

As shown in Figure 2B, the correspondence between sensory attributes and products was analyzed by PCA analysis (*p* < 0.05). It can be seen that the CT, PS, and UHP groups were located on the left side, indicating that their overall quality characteristics were similar to each other. The HTS sample was distributed on the right side, and only pungency was positively correlated with it. The UHP group was located on the left side with higher acidity, gloss, saltiness, viscosity, and fluidity, which were consistent with our QDA results (Figure 2A). In general, the sterilization had different effects on the sensory evaluation of the sauce by comparing the combined scores, and the priority order for sauce sterilization was UHP > PS > HTS.

### 3.2. Electronic Nose Analysis

**Figure 3:**
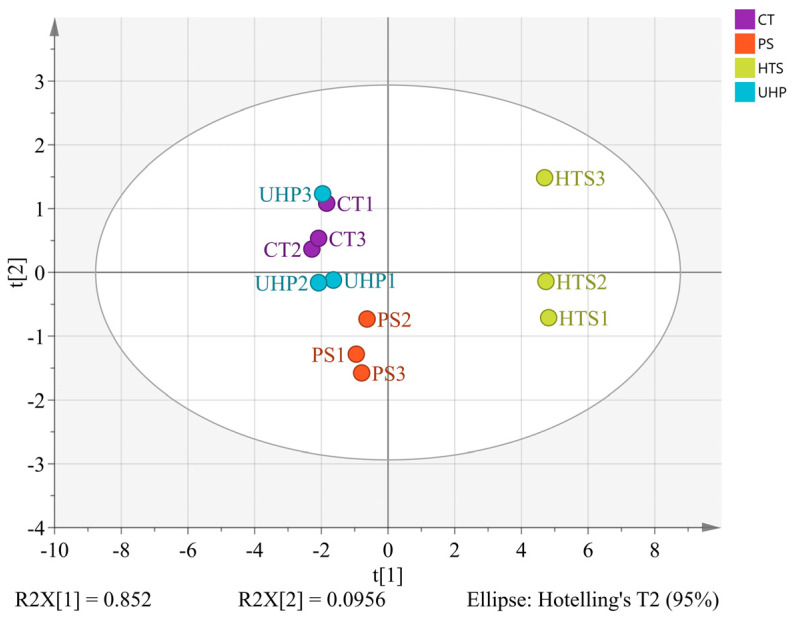
PCA bi−plot of sauces generated from electronic nose data (*p* < 0.05).

Electronic nose (e-nose) was used for rapidly discriminating the sauce treated with different sterilization methods. PCA analyses were performed to describe the difference of volatile chemical compounds. The principal component 1 (PC1) and 2 (PC2) are shown in Figure 3. According to Figure 3, the four groups were separated into three different clusters. The control and UHP groups were in the same cluster and located far away from the PS and HTS groups, suggesting that the temperature contributed significantly to volatile flavor differences. The HTS sample was positively correlated with the electronic nose sensors (W1C, W5S, W3C, W6S, W5C, W1S, W1W, and W3S), showing that the flavor intensity and volatile content of the HTS sample were richer than those of the PS, UHP, and CT samples. Thus, the sauce treated by thermal processing and non-thermal processing could be distinguished using an electronic nose combined with PCA [23]. Compared to non-thermal sterilization (UHP), the thermal sterilization easily caused the samples to undergo a Maillard reaction and resulted in a remarkable variation in the composition of volatile substances, resulting in differences in flavor [14,24]. These results provided good agreement with the classification of sensory evaluation (Figure 2).

### 3.3. Characterizing the Volatiles Compounds of the Sauce

**Figure 4:**
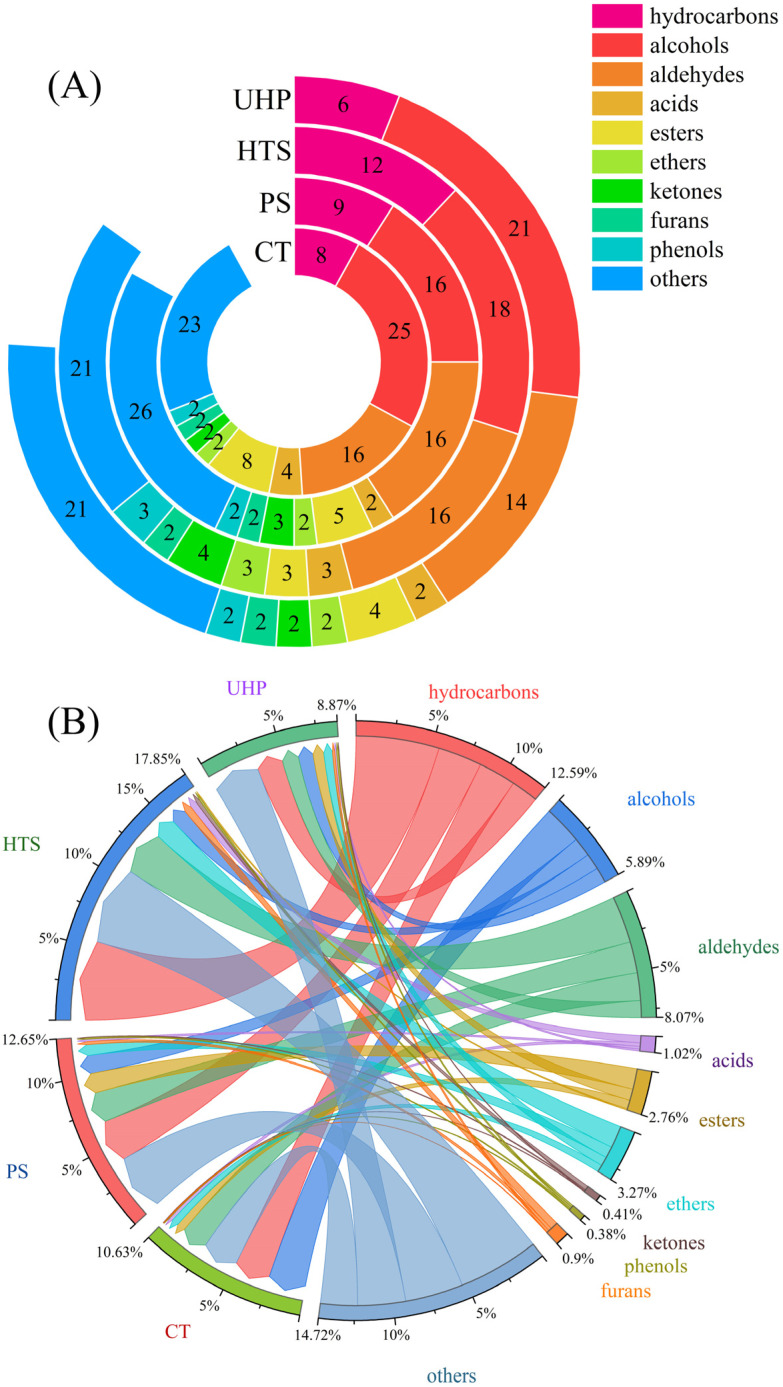
(**A**) Number of volatile compounds in the four samples (number represent the types of volatile flavor substances species); (**B**) Chord plot of volatile flavor substance contents of the four samples. The rings on the left represent the different samples. The other colored, partial loops and chords on the right represent the different volatile flavor substances. The width of the chord represents the relative contents of the volatile flavor substances.

A total of 140 volatile compounds were detected in the four samples by GC×GC-MS, which could be grouped into ten categories, including hydrocarbons, alcohols, aldehydes, acids, esters, ethers, ketones, furans, phenols, and others (Figure 4A). The volatile compounds detected from CT, PS, HTS, and UHP groups were 92, 83, 85, and 76, respectively. Liu et al. [25] also found that fresh samples are the richest in flavor species, and sterilization results in the loss of flavor species. The percentages are shown in Figure 4B, which shows that aldehydes (17.02%, 16.28%, 17.29%, 12.63%), alcohols (23.98%, 9.61%, 5.8%, 11.2%) and other compounds (22.81%, 30.27%, 29.87%, and 35.69%) were the three main components of the CT, PS, HTS, and UHP groups.

Aldehydes have low thresholds and present strong nutty or caramel notes [26]. A total of 16, 16, 16, and 14 aldehydes were detected in the CT, PS, HTS, and UHP groups, respectively, of which eleven (Hexanal [C1], 3-Furaldehyde [C2], (E)-2-Octenal [C4], Benzaldehyde [C5], Methylfurfural [C6], (E,E)-2,4-Decanedienal [C8], [Z]-2-Nonenal [C9], [E,E]-2,4-Heptadienal [C10], Octanal [C11], [Z]-3,7-Dimethyl-2,6-octenal [C14] in Table 1, Phenylacetaldehyde] were shared in the four samples. Two aldehydes (2-Hexenal [E] and Heptaldehyde) were only detected simultaneously in PS and HTS groups, which suggested that the high temperature could enhance the production of aldehydes. The contents of these 11 aldehydes had no significant difference among the four samples except Hexanal, whose content in the HTS group was significantly higher than that of the others. This result coincides with the results of the electronic nose (Figure 3), indicating that the flavor characteristics of the HTS group are significantly differentiated from the other three samples. Therefore, compared with thermal sterilization (HTS and PS groups), the UHP group exhibited more similar flavor characteristics to aldehyde compared to the control group. In addition, the contents of aldehydes in the UHP group were significantly lower than that of the others, which might be attributed to the sensitivity of aldehydes to high pressure [25].

The most critical precursors for aldehydes and esters were alcohols with a floral aroma and fruit sweetness [27,28]. In this study, 25, 16, 18, and 21 alcohols were detected in the CT, PS, HTS, and UHP samples. The contents of alcohols were decreased in all samples, and the order was CT > UHP > PS > HTS. This was because the alcohols were unstable and susceptible to changes when temperature and pressure varied. However, compared with heating treatment (PS and HTS), the type and quantity of alcohols in the UHP samples were the closest to the control samples. This result was precisely in line with the established knowledge that non-heated treatments are more suitable for retaining alcohol than heated treatments. In terms of heating treatment (PS and HTS), the types of alcohol in the PS sample were fewer than in the HTS sample, while the content was greater than that of the HTS sample. This result might be attributed to the higher decomposition or conversion of alcohols under high-temperature conditions.

Floral esters can give a unique ester flavor to a product while hiding the unpleasant taste caused by free fatty acids [29]. There were apparent differences in the quantity and concentration of esters in the four sauces (Figure 4). The number of esters was reduced by 60~90% after sterilization. Compared to the CT group (5.33%), the ester content in the PS and UHP groups increased by 101% and 54%, respectively, whereas for the HTS group, it decreased by 94%. These results indicated that temperature increase may be conducive to the formation of esters, but its efficacy may be reduced when the temperature was too high. The increased ester content in the PS and UHP groups could improve the quality of both samples, as shown in the sensory score results (Figure 2).

The aroma of the sauce was also affected by ketones, phenols, acids, terpenes, pyrazines, and furans. After sterilization, the ether, ketone, phenolic, and furan species remained unchanged and differed only in their contents. The relative contents of ketones and phenols in the HTS group were higher than in other groups. In addition, three new ketones (Acetoin, 2-Nonanone, and 4-CYCLOPENTENE-1,3-DIONE) were discovered in the HTS group, indicating that high-temperature treatment was conducive to the formation of ketones.

### 3.4. Effect of Sterilization Methods on the Volatile Compounds of Sauces

Compounds were screened from the GC×GC-MS results, which could generally divide them into groups by chemical classification, namely hydrocarbons, alcohols, aldehydes, acids, esters, ketones, ethers and others. The concentrations of each compound are shown in Table 1. Most of the four samples contained the above substances, so they were selected for further analysis of the differences in volatile compounds.

Hydrocarbon substances showed a similar smell of fruit, black pepper, and herbs. The contents changed significantly during the sterilization of the sauce, especially during thermal sterilization (HTS and PS). After HTS, the contents of A1 (Myrcene), A2 (*α*-Phellandrene), A3 (*p*-Cymenene), and A6 (*o*-Cymol) increased at least threefold. The content of A3 (*p*-Cymenene) and A4 (*α*-Curcumene) showed significant changes after UHP sterilization, and their content increased at least three times compared to the CT group. Moreover, the sweet orange and other odors of the sauce were enhanced due to the increase in the above substances, which could also contribute to a particular advantage in the sensory score, as presented in Figure 2.

In the identification results, the contents of four alcohols (B1, B3, B8, and B9) showed an apparent downward trend after sterilization. This might be due to the decomposition or transformation of those alcohols during the sterilization process. In contrast, the amount of B11 doubled after UHP treatment from 3.031 μg/kg in the CT group. Comparatively, the content of this substance decreased in the other two treatments, and it was not detected in the PS group. The contents of the other three alcohols (B5, B10, and B13) were increased. Among them, B13 (2-Nonanoll) presented the pungency flavor of pepper, which supported the pungency flavor of the sauce and also coincided with the result of the sensory score (Figure 2). B10 (Isoamylol) was not detected in the CT sample, and presented a high concentration in the PS samples (25.355 μg/kg) as well as a low content in the HTS samples (4.933 μg/kg). B5 (2-Phenethanol) and B10 (Isoamylol) provided the sauce with a floral fragrance and mild smell, which might attribute the sauce with a milder and more comfortable flavor than the CT sample after sterilization. In general, these alcohols might suffer the oxidation or esterification during high-temperature sterilization (HTS) and caused a remarkable degree of reduction, whereas the non-thermal sterilization (UHP) might protect alcohols from damage, showing the potential for alcohol flavor retention.

Aldehydes are mainly derived from the oxidation of unsaturated fatty acids. The concentration of 12 aldehyde compounds (C1, C2, C4, C5, C6, C8, C9, C10, C11, C13, C12, and C14) increased significantly after sterilization. Among them, C2 (3-Furaldehyde) was newly detected in the experiment groups. Its content in the HTS samples increased to 138.168 μg/kg. The contents of most aldehydes in the HTS group were recorded as being more than ten times higher than that of the CT group. This result was in agreement with other findings, which indicated that the rate of lipid decomposition was significantly higher with the increase in temperature and was conducive to the accumulation of aldehydes [30]. The increase in these aldehydes caused the flavor of the sauce to be more prominent and pleasant because they were satisfactory grassy and fruity compounds [31,32].

The content of esters related to fruit flavor characteristics decreased significantly after HTS, while there were increases in the PS and UHP groups compared with the CT group. Wang et al. [33] also found that the UHP-treated samples showed an increase in esters and a decrease in alcohols, indicating that the UHP conditions favor the conversion of alcohols to esters. It showed that PS and UHP treatment could promote the formation of esters. For acids, the content of D1 (Isovaleric acid) declined to about 50% in the HTS and UHP groups. Acids generally participate in esterification reactions to produce esters [34], which would play a vital role in the aroma profile of samples. The content of E1 (Ethyl phenylacetate) showed an increase after sterilization treatment, and the highest increase was observed in the UHP treatment, with a nearly twofold increase. As for the ketones, the content of G1 (Methyl hepten) in the experimental group rose by at least a factor of 1.5 comparing with the CT group (3.849 μg/kg). The previous study found that ketone, which showed fruit aromas, was unstable and could react with water through different degradation pathways to produce other kinds of ketones [35]. As their contents increases after sterilization, it could improve the odor harmony of the sauce and indirectly improve the sensory score.

**Figure 5:**
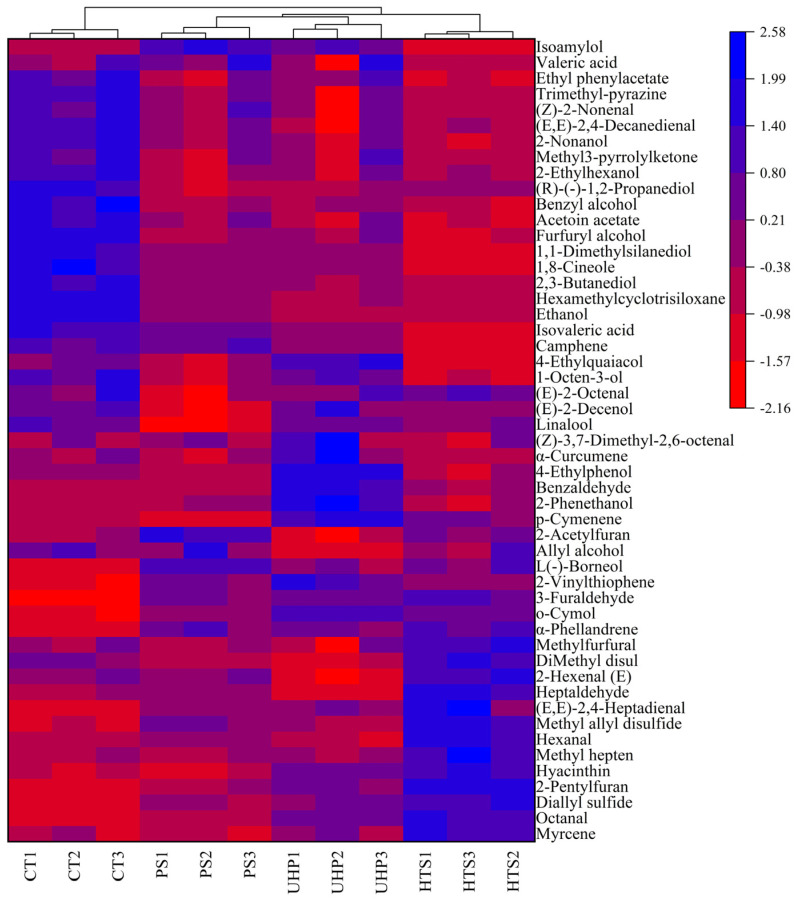
Heatmap analysis of the contents of some volatile compounds in four samples.

Regarding ethers, the contents of two ether compounds (F1 and F2, which exhibited odors of garlic, onion, and pepper) showed an upward trend after sterilization. The higher level of F1 (Diallyl sulfide, with a peppery odor) in the HTS group would alter the spicy taste compared with the CT group. It can be seen from Figure 5 that the contents of other ether compounds in the HTS group were the highest among the samples, while the UHP group was the closest to the CT group. With increasing temperature, volatile flavor compounds experienced an obvious variation compared to the fresh and UHP samples. Li et al. [30] found that the new aldehydes, ketones, hydrocarbons, alcohols, esters, and furans were detected in the heat-treated samples, suggesting that heat treatment might facilitate the transformation of these volatile substances.

### 3.5. Identification of Aroma-active Compounds by GC-O and OAV Analysis

Few volatile compounds contribute significantly to the overall aroma and sensory perception among many volatile compounds [36]. Odor activity values (OAV), calculated based on their concentration and threshold, have been widely used for estimating their contribution to aroma compounds [37]. As shown in Table 2, 17 compounds (namely 7 alcohols, 3 aldehydes, 2 esters, 2 furans, 1 ketone, 1 pyrazine, and 1 thioether) were sniffed out in the samples by GC-O analysis. Among them, the four compounds (1,8-Cineole, Linalool, Hexanal, and Dimethyl trisulfide) exhibited a more positive contribution to the aroma of the sauces. 1-Octen-3-ol, (E)-2-Octenal, and 2-Pentylfuran were also considered as the main aroma components.

The 7 odorants with an OAV range of 1–500 were effective spices in sauces (Table 2), while 10 odorants with OAVs < 1 were considered to have little effect on the overall aroma of the sauces. Among the aroma-active components, alcohols had the highest percentage (42.86%), followed by aldehydes (28.58%), furans (14.28%), and thioether (14.28%). These results also indicated that alcohols were the main aroma compounds in the sauces. According to the GC-O identification results, there were still more compounds with an OAV value less than 1, so some non-volatile flavor substances or the synergistic effect of multiple volatile flavor substances may play a larger role in flavor formation.

### 3.6. Cluster Analysis of Volatile Compounds

The 51 volatile compounds that appeared at least three times in the four samples were chosen to reflect the differences within groups. As shown in Figure 5, the 51 volatile compounds were clustered based on normalized Euclidean distance. The total contents of these volatile compounds in the PS and HTS groups were at a remarkably higher level than those of the CT and UHP groups (Table 1). Compared with the control group, there were more than double the amount of C1 (Hexanal), C6 (5-Methyl furfural), F1 (Diallyl sulfide), F2 (Methyl allyl disulfide), and I2 (2-Acetylfuran) in the PS, UHP, and HTS groups, and these substances produced aromas of fruit, grass, caramel, raw pepper, garlic, and nuts in sauces. Previous research illustrated that the furan is produced mainly by dehydrogenation or pyrolysis of sugars [3], and it may accumulate during sterilization processing. As shown in Figure 5, the samples were clearly divided into three clusters: the CT group was placed in the left cluster, the PS and UHP groups were located in the middle cluster; and the HTS group was placed in the right cluster, indicating there were significant intergroup differences of volatiles in the samples. Notably, most of the volatile compounds in heat treatment (PS and HTS) were higher than the control and UHP treatments. In particular, the contents of aldehydes showed a significant upward trend during heat treatment, indicating that the change of temperature seems to stimulate the aldehydes synthesis and alter the original aroma of the sauce. The augmenting of aldehydes during high-temperature treatment was also discovered in the study of Li et al. [30].

Among these 51 substances, 13 compounds (B2, B6, B7, B11, B13, B14, C1, C4, C5, E1, I1, I2, and J4) were sniffed out in the samples. Eight of these substances (B2, B6, B7, B11, B14, C5, E1, and I1) were found to be more abundant in the UHP group than in the PS and HTS groups, so that the flavor quality of the UHP group was classified closer to the CT group in the sensory evaluation (Figure 2) and electronic nose analysis (Figure 3). For the heat treatment, the contents of B13, I2, and J4 were higher in the PS group than in the HTS, so the flavor quality of the PS group was closer to the CT group than the HTS group in the electronic nose analysis (Figure 3). Among them, the J4 (Trimethyl-pyrazine) was mainly formed through condensation of amino ketones (formation via the Strecker decomposition between amino acids and *α, β*-butaneone) during the frying, which would further disintegrate at high temperature (HTS). The J4 mainly showed the aroma of nuts and roasted peanuts, and plays a positive role in the formation of sauce flavor [34].

### 3.7. Discrimination of Flavor Characteristics Based on Various Indicators

**Figure 6:**
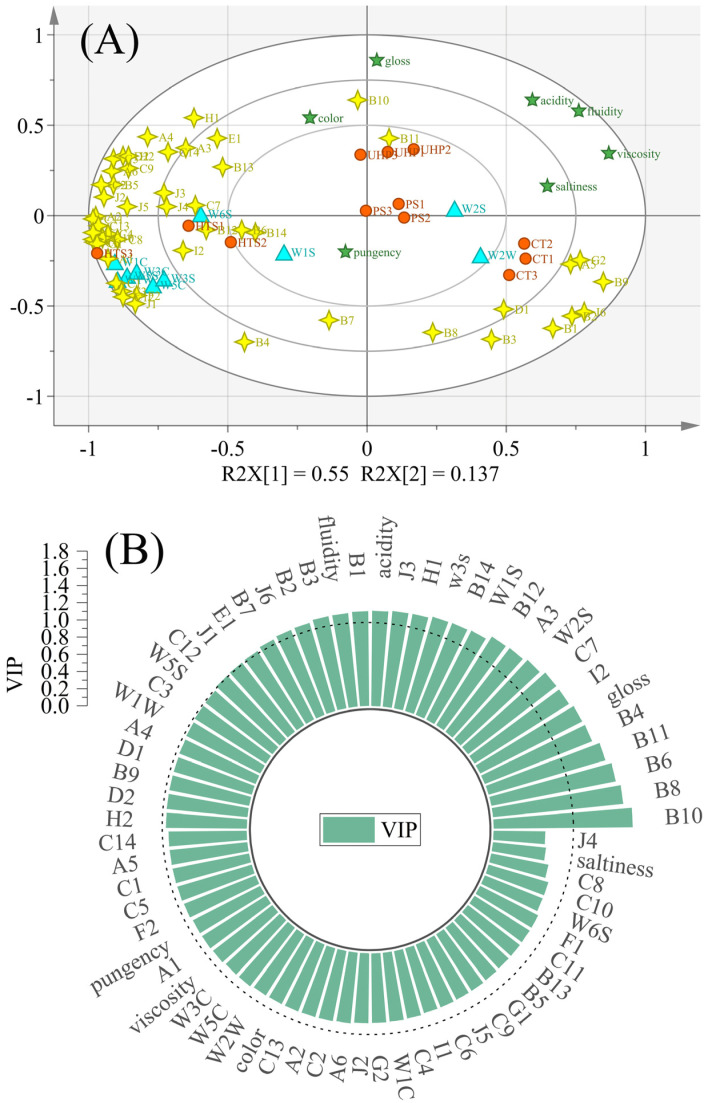
(**A**) PLS−DA analysis of each index (Orange circle: sample; Green five−pointed star: sensory evaluation index; Blue triangle: electronic nose sensor; Golden four−pointed star: some volatile compounds); (**B**) The VIP values of indicators calculated from the PLS regression model.

Electronic nose analysis, volatiles, and sensory evaluation were utilized to estimate the differences between samples by PLS-DA. The values of R^2^X, R^2^Y, and Q^2^ were 0.929, 0.986, and 0.931 respectively, which indicated that the differences among the four products could be revealed by PLS-DA [20]. As shown in Figure 6A, the HTS group was located on the left side, whereas other samples were far from this sample. This result was consistent with the results of electronic nose analysis and sensory evaluation, showing that the flavor characteristics of the HTS sample were significantly different from those of the CT, PS, and UHP groups.

The UHP group was positively correlated with color, gloss, and B10 (Isoamylol). This was in accordance with the sensory results, which showed that the UHP group scored higher in color and gloss scores than the other groups. The CT group was positively correlated with W2W, saltiness, A5 (Camphene), B1 (Ethanol), B2 (1,8-Cineole), B3 (2,3-Butanediol), B9 (1,1-Dimethylsilanediol), D1 (Isovaleric acid), G2 (Acetoin acetate), and J6 (Hexamethylcyclotrisiloxane), which was in agreement with the saltiness score as well as the volatile substance. The HTS group was positively correlated with most of the volatile flavor substances and the electronic nose sensor. It indicated that high temperature would cause a remarkable variation of volatile flavor substances, as shown in the Figure 5. By combining QDA evaluation with electronic sensory and GC×GC-MS, the chemical origin of sensory descriptions could be revealed by the PLS-DA model. As shown in Figure 6B, the variable importance for projection (VIP) values of the electronic nose sensor (W3S, W1S, and W2S), sensory evaluation index (fluidity, acidity, and gloss), and volatile compounds (A4, E1, B7, B2, B3, B1, H1, J3, B4, I2, C7, B6, and B10) were larger than 1, indicating they are the primary indexes for determining flavor characteristics.

## 4. Conclusions

This study mainly focused on the changes of sensory and aroma characteristics of sauce when it was subjected to different sterilization methods. The QDA results showed that the sensory scores in the UHP and PS groups were more similar to those of the CT group. Moreover, 140 volatile compounds were identified from samples via GC×GC-MS. The contents of volatile flavor substances increased in heat-treated samples (PS, HTS) and slightly decreased in non-heat-treated samples (UHP), indicating that the temperature arose can distinctly alter the aroma quality of the samples. The UHP and CT groups were classified into the same category in the electronic nose analysis, showing that the UHP samples were the closest to the CT samples in the comparison of the overall quality of the flavors. Furthermore, the total contents of volatile compounds in the heat-treated groups (PS and HTS) were significantly higher than for the CT group. Additionally, 7 compounds were considered to have an aromatic influence on the sauces, in which 4 compounds (1,8-Cineole, Linalool, Hexanal, and Dimethyl trisulfide) exhibited a positive contribution to the aroma of the sauces. Further in-depth research can prove beneficial to achieve the regulation of flavor substances during processing.

## Figures and Tables

**Table 1:** Concentration (μg/kg) of volatile compounds in the sauce after sterilization.

No.	Compound	CT	PS	HTS	UHP ^a^
A1	Myrcene	19.225 ± 0.253	21.754 ± 0.467	68.56 ± 0.593	25.396 ± 1.089
A2	*α*-Phellandrene	14.429 ± 1.439	58.687 ± 1.195	94.679 ± 0.892	43.084 ± 1.744
A3	*p*-Cymenene	6.332 ± 1.375	ND ^#^	41.343 ± 15.921	43.454 ± 8.84
A4	*α*-Curcumene	2.699 ± 1.659	7.161 ± 1.917	11.882 ± 1.581	10.774 ± 1.809
A5	Camphene	346.032 ± 119.564	294.264 ± 88.074	ND	129.521 ± 37.397
A6	*o*-Cymol	76.922 ± 11.96	235.538 ± 48.891	346.819 ± 52.286	247.077 ± 62.435
B1	Ethanol	140.662 ± 45.906	32.233 ± 8.803	7.053 ± 1.456	8.26 ± 1.265
B2	1,8-Cineole	225.333 ± 41.125	87.021 ± 12.099	37.214 ± 9.329	59.486 ± 13.598
B3	2,3-Butanediol	139.294 ± 44.98	72.728 ± 13.562	64.817 ± 10.991	49.047 ± 14.31
B4	Allyl alcohol	18.162 ± 2.35	21.366 ± 4.65	29.302 ± 3.605	4.693 ± 1.127
B5	2-Phenethanol	13.567 ± 2.925	19.955 ± 3.428	27.974 ± 2.813	22.145 ± 2.331
B6	Linalool	11.759 ± 2.082	ND	18.251 ± 1.457	14.294 ± 1.557
B7	Furfuryl alcohol	13.564 ± 2.409	9.837 ± 2.429	12.823 ± 1.384	10.098 ± 2.21
B8	(R)-(-)-1,2-Propanediol	19.778 ± 2.242	ND	10.708 ± 1.515	5.726 ± 1.799
B9	1,1-Dimethylsilanediol	30.907 ± 4.773	10.188 ± 1.854	ND	10.256 ± 1.685
B10	Isoamylol	ND	25.355 ± 1.89	4.933 ± 1.31	16.884 ± 1.128
B11	1-Octen-3-ol	3.031 ± 1.731	ND	2.184 ± 1.372	6.335 ± 1.395
B12	2-Ethylhexanol	2.78 ± 1.394	ND	5.633 ± 1.444	4.079 ± 1.438
B13	2-Nonanol	2.031 ± 0.836	3.282 ± 1.423	4.026 ± 0.7	3.788 ± 1.578
B14	Benzyl alcohol	1.841 ± 0.92	ND	2.88 ± 1.347	2.269 ± 0.983
C1	Hexanal	39.014 ± 8.473	94.861 ± 16.756	245.455 ± 62.34	30.105 ± 4.762
C2	3-Furaldehyde	ND	83.814 ± 13.866	138.168 ± 30.258	72.846 ± 13.998
C3	Heptaldehyde	7.621 ± 1.441	23.502 ± 5.071	70.486 ± 14.993	ND
C4	(E)-2-Octenal	4.976 ± 1.867	6.257 ± 1.908	17.556 ± 3.935	9.061 ± 1.618
C5	Benzaldehyde	7.279 ± 1.454	12.626 ± 1.517	21.744 ± 2.451	17.696 ± 1.917
C6	Methylfurfural	4.04 ± 1.354	9.911 ± 1.966	22.758 ± 2.665	8.627 ± 2.277
C7	(E)-2-Decenol	5.158 ± 1.669	ND	12.687 ± 1.953	9.579 ± 1.453
C8	(E,E)-2,4-Decanedienal	2.895 ± 0.869	4.639 ± 1.383	8.017 ± 1.752	4.327 ± 1.541
C9	(Z)-2-Nonenal	ND	2.628 ± 1.436	4.674 ± 1.346	3.142 ± 1.421
C10	(E,E)-2,4-Heptadienal	9.115 ± 2.102	21.593 ± 3.44	43.182 ± 13.778	18.107 ± 1.582
C11	Octanal	8.486 ± 1.453	20.545 ± 3.157	52.979 ± 10.234	24.639 ± 4.104
C12	2-Hexenal (E)	3.583 ± 1.381	10.323 ± 1.742	30.29 ± 4.059	ND
C13	Hyacinthin	4.57 ± 1.333	9.762 ± 1.973	47.242 ± 9.864	19.307 ± 3.058
C14	(Z)-3,7-Dimethyl-2,6-octenal	19.674 ± 1.874	25.532 ± 1.347	34.466 ± 3.189	29.824 ± 4.037
D1	Isovaleric acid	45.522 ± 12.588	35.814 ± 6.488	23.574 ± 6.401	24.677 ± 3.587
D2	Valeric acid	ND	7.18 ± 1.529	9.599 ± 1.61	6.589 ± 1.764
E1	Ethyl phenylacetate	2.287 ± 1.337	3.247 ± 1.743	5.039 ± 1.873	6.18 ± 1.329
F1	Diallyl sulfide	50.563 ± 8.873	108.325 ± 17.905	247.839 ± 47.325	102.679 ± 23.25
F2	Methyl allyl disulfide	75.27 ± 15.856	94.173 ± 13.776	149.808 ± 36.008	62.935 ± 13.382
G1	Methyl hepten	3.849 ± 1.24	10.084 ± 1.96	32.38 ± 8.886	10.926 ± 2.341
G2	Acetoin acetate	5.415 ± 1.099	2.492 ± 1.362	ND	2.366 ± 1.348
H1	4-Ethylquaiacol	5.787 ± 1.254	8.117 ± 2.486	11.791 ± 2.238	13.579 ± 2.268
H2	4-Ethylphenol	10.198 ± 1.646	13.397 ± 1.653	21.209 ± 1.727	18.731 ± 2.662
I1	2-Pentylfuran	7.862 ± 1.87	33.258 ± 8.989	114.378 ± 21.602	39.217 ± 5.01
I2	2-Acetylfuran	1.95 ± 0.829	22.821 ± 3.273	20.444 ± 3.135	2.843 ± 1.871
J1	DiMethyl disul	16.87 ± 2.024	14.791 ± 2.659	39.654 ± 8.906	11.475 ± 1.434
J2	2-Vinylthiophene	12.665 ± 1.453	26.068 ± 3.248	34.628 ± 5.479	23.231 ± 2.87
J3	L(-)-Borneol	ND	43.117 ± 9.886	45.019 ± 4.566	19.815 ± 2.541
J4	Trimethyl-pyrazine	2.015 ± 0.795	3.207 ± 1.476	4.677 ± 1.244	3.418 ± 1.566
J5	Methyl3-pyrrolylketone	2.371 ± 1.095	3.06 ± 1.201	7.276 ± 1.501	4.85 ± 1.594
J6	Hexamethylcyclotrisiloxane	22.686 ± 3.634	6.266 ± 1.46	ND	3.362 ± 1.354
Total		1470.069 ± 370.786	1660.779 ± 327.338	2308.1 ± 425.314	1320.799 ± 271.657

^a^ Note: CT, PS, HTS, and UHP refers to the control group, pasteurized, high-temperature sterilization and ultra-high-pressure treatment, respectively. ^#^ The compound was not detected.

**Table 2:** Odor activity value of the aroma compounds in sauces.

Compounds	CAS	RI	OAV				Odor Quality
			CT	PS	HTS	HHP	
esters							
Ethyl benzoate	93-89-0	1577	ND	ND	<1	<1	Floral, fruity
Ethyl phenylacetate	101-97-3	1690	<1	<1	<1	<1	Honey, rose
elcohols							
1,8-Cineole	470-82-6	1162	204.85	79.11	33.83	54.08	Eucalyptus, minty, balsamic
1-Octen-3-ol	3391-86-4	1386	2.02	ND	1.46	4.22	Mushroom-like
2-Nonanol	628-99-9	1455	<1	<1	<1	<1	Floral
Linalool	78-70-6	1478	36.75	ND	57.03	44.67	Sweet, flower
Furfuryl alcohol	98-00-0	1566	<1	<1	<1	<1	Burnt sugar
Benzyl alcohol	100-51-6	1766	<1	ND	<1	<1	Sweet, flower
Phenethyl alcohol	60-12-8	1801	<1	<1	<1	<1	Rose-like, floral
aldehydes							
Hexanal	66-25-1	1025	7.80	18.97	49.09	6.02	Cut grass, green
(E)-2-Octenal	2548-87-0	1364	1.66	2.09	5.85	3.02	green leaf
Benzaldehyde	100-52-7	1437	<1	<1	<1	<1	Roasted nuts
ketones							
2-Nonanone	821-55-6	1331	ND	<1	<1	ND	Fruits-like, flowers-like
furans							
2-Pentylfuran	3777-69-3	1179	1.36	5.73	19.72	6.76	Bean, fruit, green
2-Acetylfuran	1192-62-7	1419	<1	<1	<1	<1	Roasted, smoky
pyrazines							
Trimethyl-pyrazine	14667-55-1	1339	<1	<1	<1	<1	Roast
thioether							
Dimethyl trisulfide	3658-80-8	1306	75.71	122.23	382.09	116.18	Mint smell, fresh onion-like

## Data Availability

The data and materials supporting the conclusions of this study are included within the article.

## Abbreviations

PXDB	Pixian Douban
CT	control
PS	pasteurized
HTS	high-temperature sterilization
UHP	ultra-high-pressure treatment
HS-SPME	headspace solid-phase microextraction
GC×GC-MS	comprehensive two-dimensional gas chromatography-mass spectrometry
GC-O	gas chromatograph-olfactometry
IS	internal standard
NIST	National Institute of Standards and Technology
OAV	odor activity value
QDA	quantitative descriptive analysis
PLS-DA	partial least squares discriminant analysis
PCA	principal component analysis
